# Comprehensive Analysis Reveals the Genetic and Pathogenic Diversity of *Ralstonia solanacearum* Species Complex and Benefits Its Taxonomic Classification

**DOI:** 10.3389/fmicb.2022.854792

**Published:** 2022-05-06

**Authors:** Ruimei Geng, Lirui Cheng, Changdai Cao, Zhengwen Liu, Dan Liu, Zhiliang Xiao, Xiuming Wu, Zhenrui Huang, Quanfu Feng, Chenggang Luo, Zhiqiang Chen, Zhenchen Zhang, Caihong Jiang, Min Ren, Aiguo Yang

**Affiliations:** ^1^Key Laboratory for Tobacco Gene Resources, Tobacco Research Institute, Chinese Academy of Agricultural Sciences, Qingdao, China; ^2^Shandong Rizhao Tobacco Company Ltd., Rizhao, China; ^3^Key Laboratory of Crop Genetic Improvement, Engineering and Technology Research Center for Tobacco Breeding and Comprehensive Utilization of Guangdong, Crops Research Institute of Guangdong Academy of Agricultural Sciences, Guangzhou, China

**Keywords:** *Ralstonia solanacearum*, genetic, diversity, pathogenicity, pangenome

## Abstract

*Ralstonia solanacearum* species complex (RSSC) is a diverse group of plant pathogens that attack a wide range of hosts and cause devastating losses worldwide. In this study, we conducted a comprehensive analysis of 131 RSSC strains to detect their genetic diversity, pathogenicity, and evolution dynamics. Average nucleotide identity analysis was performed to explore the genomic relatedness among these strains, and finally obtained an open pangenome with 32,961 gene families. To better understand the diverse evolution and pathogenicity, we also conducted a series of analyses of virulence factors (VFs) and horizontal gene transfer (HGT) in the pangenome and at the single genome level. The distribution of VFs and mobile genetic elements (MGEs) showed significant differences among different groups and strains, which were consistent with the new nomenclatures of the RSSC with three distinct species. Further functional analysis showed that most HGT events conferred from *Burkholderiales* and played a great role in shaping the genomic plasticity and genetic diversity of RSSC genomes. Our work provides insights into the genetic polymorphism, evolution dynamics, and pathogenetic variety of RSSC and provides strong supports for the new taxonomic classification, as well as abundant resources for studying host specificity and pathogen emergence.

## Introduction

*Ralstonia solanacearum* is a soilborne Gram-negative bacterium that is responsible for lethal wilting in plants globally, which is known as bacterial wilt disease (Salanoubat et al., 2002). It had a wide geographical distribution and extremely diverse host range, involving more than 450 plants belonging to over 50 botanical families (Kumar et al., 2018). In addition, *Ralstonia solanacearum* has a high pathogenic diversity, which could exhibit diverse virulence under different temperatures (Denny, 2007; Li et al., 2016). *Ralstonia solanacearum* causes extensive damage and is considered to be one of the most destructive plant diseases in the world (Elphinstone, 2005). Traditionally, *R. solanacearum* is divided into five races and six biovars according to host range and biochemical characteristics (Denny and Hayward, 2001). At present, *R. solanacearum* is considered as a heterogeneous species with multiple genetic groups, showing a high degree of genetic diversity among different strains, which are collectively referred to as the *R. solanacearum* species complex (RSSC; Genin and Denny, 2012). Based on geographical origin, RSSC could be classified into four taxonomic levels with species, phylotypes, sequevars, and clones, and four phylotypes were identified by multiplex-PCR as I (Asia), II (America), III (Africa and India Ocean), and IV (Indonesia). With sequences alignment and phylogenetic analysis, RSSC were divided into three species *R. pseudosolanacearum*, *R. solanacearum*, and *R. syzygii*, and further classified *R. syzygii* into three subspecies as *R. syzygii* subsp. *syzygii*, *R. syzygii* subsp. *indonesiensis*, and *R. syzygii* subsp. *celebesensis* (Remenant et al., 2010; Safni et al., 2014). With the development of next-generation sequencing technologies, an increasing number of “*R. solanacearum*” genome sequences [current taxonomy in the National Center for Biotechnology Information (NCBI)] have been recently sequenced, which will facilitate further genomic analysis and taxonomic classification of RSSC.

The development of bacterial wilt depends on the pathogenicity and virulence of RSSC, which is cumulative and complex, and usually involves multiple VFs (Schell, 2000). For example, exopolysaccharide (EPS) is the main virulence factor, which enhances the rate and extent of infection from root to stem, and is inferred to limit the transportation of water from xylem vessels, resulting in wilting of the host plant (Annett et al., 2011; Hayashi et al., 2019). Type IV pili (T4P) is a basic virulence factor of many bacteria. It has a range of functions, such as biofilm formation, adhesion, twitching motility, conjugate DNA transfer, and phage infection (Craig et al., 2019). T4P was found to be an essential virulence factor for biofilm formation of the RSSC in infecting potato (Wairuri et al., 2011). Macromolecular secretion systems play an important role in secretory proteins and pathogenicity (Sophie and Eduardo, 2017). Several macromolecular secretion systems have been found in the RSSC, including type I secretion systems (T1SS), T2SS, T3SS, T4SS, T5SS, and T6SS. The efflux genes *acrA/B* in T1SS have been shown to be necessary to cause infection of RSSC (Alav et al., 2021). An enzyme secreted by T2SS could degrade the cell wall, and pectinase decomposes pectin to form oligosaccharides as the substrate for bacterial growth (Tsujimoto et al., 2008). T3SS was considered to be the main virulence factor of RSSC, which could inject many effector proteins into plant cells, resulting in host disease or hypersensitivity of resistant plants. T4SS can translate DNA, proteins, or other macromolecules to target bacteria or eukaryotic cells, and plays an important role in motility, biofilm formation, and virulence in plant pathogens (Alvarez-Martinez and Christie, 2009; Ogunyemi et al., 2019). T5SS is known for its cell surface adhesion function and could be divided into five subtypes, which plays a great role in diverse bacterial pathogenicity (Leo and Linke, 2012). T6SS is considered to be involved in pathogenesis, bacterial interactions, and inter-bacterial competition and has also been found to play an important role in the virulence of RSSC (Asolkar and Ramesh, 2020; Smith et al., 2020). These different VFs were detected in each strain of this study, indicating that the pathogenicity of RSSC was closely related to their VFs.

Horizontal gene transfer (HGT), also known as lateral gene transfer, is usually defined as the transfer of genetic information between organisms without mating (Soucy et al., 2015). HGT is also considered to be a major evolutionary driver that plays an integral role in causing different pathogenicity of distant strains, as well as the diversification, adaptation, and speciation (Ochman et al., 2000; Nakamura et al., 2004; Arnold et al., 2021). For RSSC, HGT occurred easily in all phylotypes of these bacteria and played a key role in the rapid evolution of its pathogenicity (Coupat-Goutaland et al., 2010). For example, two novel type III effectors were thought to have been obtained through HGT in *R. solanacearum* strain HA4-1, and these effectors might be related to host specificity and pathogen evolution (Tan et al., 2019). Studies have shown that the transfer of the *imuABC* DNA polymerase gene from *Cupriavidus taiwanensis* could accelerate the adaptation of *R. solanacearum* to new hosts (Remigi et al., 2014). Evidence from these studies has proved that HGT plays a significant role in the evolution, pathogenicity, and adaptation of the RSSC. Nevertheless, the mechanisms underlying genetic exchange are difficult to determine. Here, we attempted to detect all of HGT events in these sequenced genomes to explore the functions of HGT in the RSSC.

In this study, we conducted a pangenomic analysis with 131 complete genome sequences of the RSSC, focusing on the genetic diversity, phylogenetic relationships, and pathogenicity in these strains. Pangenome analysis can identify core and accessory genomes in the genus or among populations, which could reveal key differences in their evolutionary histories (Brockhurst et al., 2019; Rosselli et al., 2021). We conducted phylogenetic analyses at the core genome, pangenome and whole-genome levels and found that RSSC could be clearly divided into three different species. All HGT events of the RSSC were detected and functional analysis was performed to study the evolution and speciation of these strains. Based on the pangenome and single genome level analyses, we identified the prophages, genomic islands (GIs), insertion sequence (IS), and VFs to explore the genetic and pathogenic diversity of different species in RSSC. This study highlights the advantages of pangenomic analysis for inferring the evolution and reveals the genomic diversity and pan-VFs of different strains, which is helpful in pathogenicity and host specificity of RSSC in the future.

## Materials and Methods

### Genome Sequencing Data and Annotation of RSSC

To achieve a comprehensive overview of the RSSC, the present study included all the complete genome sequences (included the complete and chromosome-level sequences) of the RSSC available in the genome database of the NCBI. The genome integrity was assessed using the Benchmarking Universal Single-Copy Orthologs (BUSCO v5.2.2) software (Waterhouse et al., 2017). Prokka software was used to obtain the protein and coding DNA sequences (CDS) of each strain so as to ensure the consistency and reliability of annotation and gene prediction (Seemann, 2014). Supplementary Table S1 summarizes the original genome sequences used in this study.

### Average Nucleotide Identity Analysis

To explore the genetic distance and relatedness among these RSSC strains, Average Nucleotide Identity (ANI) analysis was performed using the python program “pyani v0.2.7” (Pritchard et al., 2016). The analysis was based on the MUMER (v3.23) algorithm on the local computer with the default setting (Kurtz et al., 2004). The heatmap software package (v1.0.12) in R was used to deliver the graphical heat map and dendrogram for ANI values.

### Pangenome Analysis

The pipeline of Roary (v3.13.0) was used to determine the ortholog groups with the default parameters (Page et al., 2015; Sitto and Battistuzzi, 2020). The determination and selection of core gene thresholds are critical to the accuracy of pangenome analysis. Considering the sequence data were relatively complete genome assemblies, a core threshold (99% ≤ strains ≤100%) was used instead of a soft-core threshold (95% ≤ strains ≤99%), which means the core genes could be shared by more than 129 strains of the RSSC used in this study. The expansiveness of the pangenome was estimated in R software with the “micropan” package according to Heap’s law model (Snipen and Liland, 2015). The decay parameter alpha was calculated with 5,000 random permutations.

### VF Analysis in the Pangenome and in Each Strain

To obtain insights into the virulence and potential pathogenicity of different strains and species in the RSSC, the distribution of VFs in the pangenome and in each single genome were explored using the Virulence Factors Database (VFDB; Liu et al., 2018). For the genome sequences of each strain and the pangenome, the search was performed using the program BLASTX 2.6.0+, which uses nucleotide versus protein alignments based on the punctuation matrix BLOSUM62. The BLAST search was carried out against all the virulence-associated proteins adhering to the following parameters: Bit score > 100, E-value <1.0 e-5, coverage >50%, and percentage identity >30%.

### Phylogenetic and Evolutionary Analysis

To determine the species relationship among the RSSC strains, the core and pangenome phylogenetic analysis was based on the gene families of Roary. Mafft (v7.310) was used to build a sequence alignment (Katoh and Standley, 2013), and FastTree (v1.0) was used to build a concatenated tree using the neighbor-joining method with default parameters (Price et al., 2009). CDS of orthologs were codon aligned by PAL2NAL (v14.0) following their proteins (Suyama et al., 2006). Subsequently, the codon alignments and phylogenetic trees obtained in the above step were subjected to a series of evolutionary selection tests by Hypothesis testing using Phylogenies (HyPhy) with the Fast Unconstrained Bayesian AppRoximation (FUBAR) method (Pond et al., 2004).

### HGT Detection

For HGT detection, the RSSC genomes were analyzed for recent transferred acquired genes using HGTector (v2.0b2) with the alignment method of double index alignment of next-generation sequencing data (diamond; Zhu et al., 2014; Buchfink et al., 2015). To detect potential horizontally transferred genes, the self-groups *Ralstonia* (rank genus with taxon ID 48736) and *Burkholderiales* (rank order with taxon ID 80840) were downloaded from the NCBI. Each RSSC genome was searched against this database using an E-value of 1e-5, a minimum identity threshold of 50%, and a minimum coverage of 50%. The potential donors and HGT genes were extracted from the output files.

### MGEs Analysis

MGEs can mediate DNA acquisition and promote the transmission of genetic material between different bacteria (Ochman et al., 2000; Yin et al., 2020). To detect the distribution of MGE in the RSSC pangenome, gifrop (v0.0.9)^1^ was used to help to identify GIs based on the results of Roary. ISEScan (v1.7.2.3) was used to identify IS elements in each genome (Xie and Tang, 2017). In addition, PhiSpy software (v4.2.19) was used to predict prophages in each strain (Akhter et al., 2012).

### Functional Annotation

To detect the function of the pangenome, the sequences of core genome, accessory genome, and unique genes were searched to compare the distribution of their functional categories using eggNOG (Huerta-Cepas et al., 2018). Gene ontology (GO) enrichment analysis of these proteins was performed using Web Gene Ontology Annotation Plot (WEGO v2.0) database (Ye et al., 2018). Functions of the genes of HGT events in the eggNOG database were also investigated.

## Results and Discussion

### Genomic Relatedness and Characterization of the RSSC

**Figure 1:**
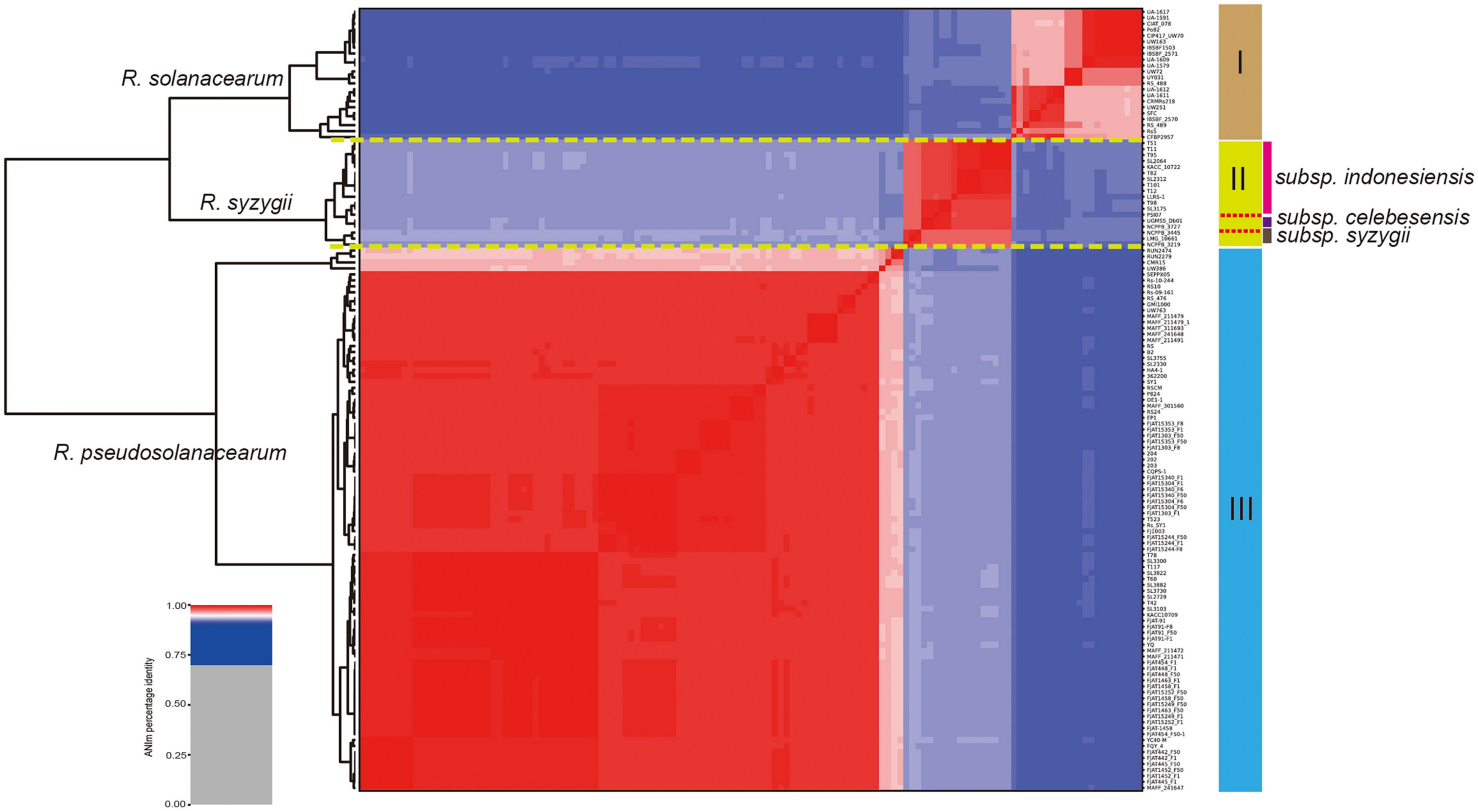
Taxonomic classification of *Ralstonia solanacearum* species complex (RSSC) based on comparison at the whole genome level. Phylogenetic tree based on blast all vs. all of 131 RSSC strains, which could be divided into three species, and the species names were labeled on the front of each branch (numbers I, II, and III were marked in the right). Three stains belong to the subspecies of *R. syzygii* subsp. *indonesiensis* were marked with three different colors near their names.

In this study, a total of 137 complete genome sequences of *R. solanacearum*, *R. syzygii*, and *R. pseudosolanacearum* strains (taxonomy ID: 305, 28,097, and 1,310,165) collected from different geographic locations and isolation sources were preliminarily analyzed (Supplementary Table S1). However, in other studies, several strains were found to be incomplete or missing megaplasmids from assembly files (e.g., Rs-09-161, Rs-10-244, T110, T12, CFBP2957, and T25; Sabbagh et al., 2019). To confirm the genome integrity, we used BUSCO to analyze these genomes by searching a total of 124 BUSCO groups (bacteria_odb10). In order to improve the quality of RSSC pangenome and cover more strains, the complete metric (C%) was set at 95%. Finally, it was found that the completeness of six strains was poor, and then they were excluded in the subsequent analysis (Supplementary Table S1). To be consistent with the genomic data, all sequences were annotated using Prokka (Seemann, 2014). The correct taxonomic classification is essential for obtaining high-quality pangenomes (Wu et al., 2020). To determine the taxonomic status and obtain a high-quality pangenome of *R. solanacearum*, ANI values were calculated to estimate the genetic relatedness among strains. ANI has become one of the main genome options for DNA–DNA hybridization for taxonomic purposes (Arahal, 2014). The ANI value of *R. solanacearum* was observed to range from 91.45% to 99.99%, and it could be divided into three groups (Figure 1). Of these strains, 83.2% (109 of 131) had ANI values of less than 95.0%, reflecting the great genetic distances between these strains. Furthermore, there were obvious differences in similarity among the three groups. Groups I and II had s similarity of 92.2%; groups I and III, of 91.7%; and groups II and III, of 92.9%. However, the similarity among strains in the same group was greater than 95%. The similarities of 22 strains in group I were 96.1%–99.9%, those of 18 strains in group II were 97.7%–99.9%, and those of 91 strains in group III were 96.0%–99.9%, with average similarity values of 97.6%, 98.8%, and 99.0%, for groups I, II, and III, respectively. The previously suggested species threshold of 95% ANI can represent the same species (Goris et al., 2007). Thus, these 131 RSSC strains could be divided into three different species: *R. solanacearum* (I), *R. syzygii* (II), and *R. pseudosolanacearum* (III) and then regarded as a species complex. These results were also consistent with the previous taxonomic studies of RSSC (Remenant et al., 2010; Safni et al., 2014), and helped to obtain the correct classification of most strains in this study (103 out of 131). The GC content, genome size, and number of CDS for all the analyzed genomes were determined (Supplementary Table S1). The length of the obtained RSSC genomes ranged from 3,417,386 bp (strain CFBP2957) to 6,147,432 bp (strain T78), which equals a difference of approximately 44.4%. The average ORF count was found to be 5,045 ORFs, and the lowest and the highest numbers of ORFs were 3,218 (strain: CFBP2957) and 6,349 (strain: T110), respectively, and the difference between them was approximately 49.3%. Meanwhile, there were also significant differences in the CDS, proteins, and genome sizes among different species. For example, the average numbers of CDS of *R. solanacearum*, *R. syzygii*, and *R. pseudosolanacearum* were 4,623, 4,836, and 5,178, respectively, with a difference of 12.0% between the maximum and minimum numbers. However, there were no obvious differences in GC content among the three species. These vast differences supported the high complexity and genomic diversity of RSSC strains.

### Construction and Analysis of the RSSC Pangenome

**Figure 2:**
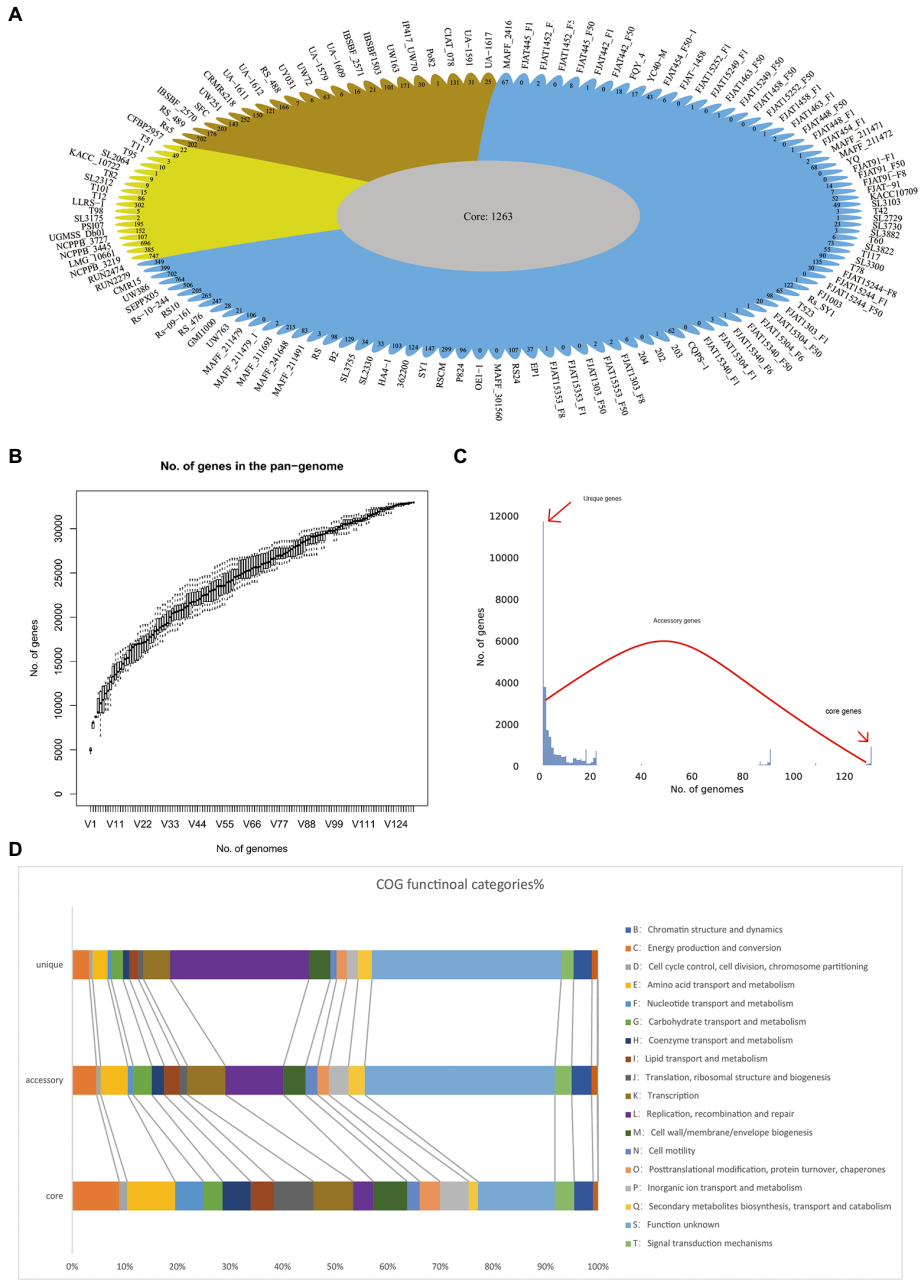
Pangenome shape and function of RSSC. **(A)** The pangenome flower plot showing the core genome and different unique gene for each strain, the different colors correspond with the different groups in the core genome tree. **(B)** Gene accumulation curves for the pangenome, the cumulative sizes were calculated by selecting strains without replacement in random order 1,000 times. **(C)** A histogram of the prevalence of different gene families in the pangenome. A total of 30,763 non-redundant gene families identified in 131 genomes are based on their frequency distribution. Three gene categories are clearly distinguished, highlighting the unique genes (genes that only exist in one strain), core genes (gene families are present in more than 120 strains), and accessory genes (gene families that exhibit variable frequencies). **(D)** Distribution of COG categories in the core, accessory, and unique genes.

To determine the genomic composition of RSSC, 131 genomes with a high level of completeness, were used for the construction and analysis of its pangenome. In this study, 32,961 non-redundant groups from the RSSC pangenome were identified, including 1,263 core gene families, 19,911 accessory gene families, and 11,787 unique genes, which were found across more than 129 genomes, in more than two genomes, and in a single genome, respectively (Figure 2A; Supplementary Table S2). Based on Heaps’ law model, we obtained an alpha value less than 1.0, which exhibited an open pangenome structure for the gene accumulation curve of the RSSC (Figure 2B). Due to the existence of an expanded library of dispensable genes from different strains, the pangenome was relatively large. The proportion of dispensable genomes (96.2%), including accessory gene families (60.4%) and unique genes (35.8%), was far more than that of core gene families (3.8%), which reflected the extremely high genetic diversity among strains of the RSSC (Figure 2C). The distribution of accessory genes was diverse across different strains, varying from 1,773 genes (CFBP2957) to 4,011 genes (FJAT-1458), which was approximately a 2.32-fold difference. Strain CFBP2957 belonged to phylotype IIA and showed certain species specificity of tomato. FJAT-1458 was thought to be a candidate strain to develop bacterial wilt vaccine (Chen et al., 2017). Fourteen strains had huge accessory families that accounted for more than 76.0% of their whole genomes. Similarly, unique genes differed significantly among these strains. Strain NCPPB_3445 had the highest number of unique genes (696), which accounted for 16.7% of its whole genome, whereas 20 other strains had no unique genes. These results indicate that a large and uncertain additional genome is needed to identify all the genes accessible to this species, ensuring that the RSSC responds quickly to various environments or hosts. Clusters of Orthologous Groups of proteins (COGs) analysis suggested that the accessory and unique genes are abundant in replication, recombination, and repair (Figure 2D). Thus, these diverse accessory and unique genes may be responsible for the high pathogenicity and the potential host-specification of these strains.

To better explore the genetic diversity among different species in RSSC, we also conducted sub-pangenome analysis for the three species. The sub-pangenome size of the three groups differed considerably, with 10,416, 10,294, and 18,442 nonhomologous gene families in *R. solanacearum*, *R. syzygii* and *R. pseudosolanacearum*, respectively (Supplementary Figure S1; Supplementary Table S2). These sub-pangenomes were far fewer than that of the RSSC pangenome, which contained 32,961 nonhomologous gene families (Supplementary Figure S1A). In the sub-pangenome of *R. pseudosolanacearum*, the dispensable gene families (accessory genomes and unique genes) accounted for 79%, and its accessory genome was the largest among the three groups (Supplementary Figure S1B). The size of the sub-pangenome of *R. solanacearum* was the smallest, while its core genome was the largest (30.2%) of the three groups (Supplementary Figure S1C). Compared with *R. solanacearum* and *R. syzygii*, *R. pseudosolanacearum* had the largest sub-pangenome but the smallest core genomes (21.0%; Supplementary Figure S1D). Further analysis showed that there were 14,627 gene families, including 1,719 core gene families, 6,640 accessory families and 6,268 unique genes appearing only in the sub-pangenome of *R. pseudosolanacearum*, but not in *R. solanacearum* and *R. syzygii*. Similarly, 6,616 gene families (including 893 core, 2,928 accessory and 893 unique genes) only existed in *R. syzygii*, and 7,493 gene families only occurred in *R. solanacearum* (including 1,193 core, 3,576 accessory, and 2,724 unique genes). Hence, so many genes only appear in a single species, which may be associated with its special traits, and could a good gene resource to study the pathogenicity and host specificity of this species. These obvious differences in the pangenome of the RSSC and sub-pangenome of three groups indicated tremendous diversity between the three species.

### Phylogenetic Analysis of the RSSC

**Figure 3:**
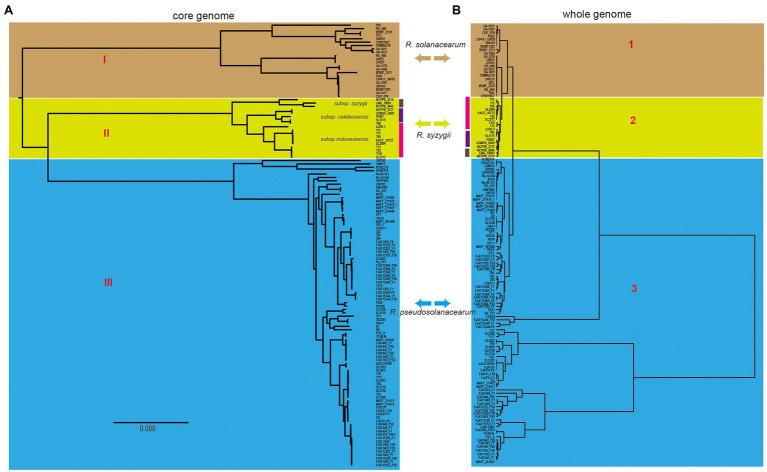
Comparison of two phylogenetic trees constructed using core genome and whole genome, respectively. **(A)** Core genome tree was based on 1,129 core genes of 131 strains, and different cores represent three different species, which was linked with that in the whole genome tree (right). **(B)** Whole genome tree could also be divided into three groups, the colors linked with that in core genome tree (left). Bidirectional arrows in the middle represents the same species in these two trees.

Phylogenetic analysis was performed for all the included RSSC strains to understand the pattern of evolution. Using the pangenome and core-genome alignment concatenation approach, phylogenetic trees were built up for the set of 131 genomes, and the constructed phylogenetic trees showed high phylogenetic diversity and clear evolutionary relationships among these strains. Based on the topological structure and evolutionary distances, the core-genome tree could be divided into three groups (Figure 3A), which is consistent with the three species in the whole-genome tree based on the ANI identity. The distribution of strains in three groups were same as those in the whole-genome tree (Figure 3B). In group I (*R. solanacearum*) of the core-genome tree, strain RS5 showed a different evolution distance with other 21 strains. This strain was isolated from tomato and found to cause no disease on tobacco and little or none on pepper (Ji et al., 2007). There were 702 unique genes in RS5, which was the largest in *R. solanacearum*, with an average of 131 unique genes in this branch. These unique genes may cause RS5 to show different pathogenicity to the hosts. The strains UW386, CMR15, RUN2279, and RUN2474 of *R. pseudosolanacearum* displayed a different evolution relationship with the other 83 strains. All four strains belong to phylotype III, although they were isolated from different environments or hosts. For example, UW386 was isolated from the soil; RUN2279 and RUN2474, from potatoes; and CMR15, from tomato (Steidl et al., 2021). CMR15 is thought to be the only *R. pseudosolanacearum* strain with the *ripG8* gene, which facilitates co-evolution with specific host targets such as *Medicago truncatula* (Wang et al., 2016). Similar to these strains in group III (*R. pseudosolanacearum*) of the whole-genome tree, phylotype III strains could be clustered together with the core-genome and whole-genome alignment, which could be helpful for the phylotype classification of the RSSC. Here, the phylogenetic analysis based on core-genome alignment was consistent with that of the whole-genome tree, and could accurately distinguish the different species of the RSSC. This provides a new perspective for the classification of RSSC and other plant pathogens, because core-genome alignment was considered to be a potential standard for bacterial taxonomy (Chung et al., 2018).

To further explore the phylogenetic relationship, a pangenome tree was also constructed, which showed a high phylogenetic diversity. The pangenome tree could be divided into four groups (Supplementary Figure S2) and showed a similar relationship with the core phylogenetic tree. For example, group A contained five strains, including RS, CRM15, RUN2279, RUN2474, and Rs-09-161, which differed significantly from the other strains of the same species of *R. pseudosolanacearum* in the core-genome tree. We found that off the 131 strains, these five strains in group A had the largest number of unique genes. The dispensable genomes of these strains accounted for more than 76% of their genomes, which were larger than other strains. Rs-09-161 belonged to phylotype I, which was isolated from eggplant in India (Ramesh et al., 2014). The strain is thought to be rich in VFs and type III effectors (T3Es), including two unique effectors, which may be related to the host specificity (Asolkar and Ramesh, 2018). Interestingly, strains from groups C and D were consistent with those in groups I (*R. solanacearum*) and III (*R. pseudosolanacearum*) of the core-genome tree, respectively. The combination of strains in groups A and D was nearly equivalent to group III (*R. pseudosolanacearum*), although they were located far in the pangenome tree, suggesting that *R. pseudosolanacearum* might had higher genetic polymorphism than other two species in the RSSC. In group C, strains SEPPX05 and UW386 were significantly different from other 22 strains. SEPPX05 was isolated from sesame and belonged to phylotype I. It had a highly conserved secretion system that regulates interactions with the host (Li et al., 2018). Further analysis showed that the dispensable genomes of UW386 (73.4%) were closer to the strains of *R. syzygii* (with an average of 73.0%), but it had an obvious difference compared to other strains of *R. pseudosolanacearum* (with an average of 75.0%). Hence, we suspect that the close lineages in the pangenome tree shared more accessory and unique genes, which could lead to differences in the evolution process. In conclusion, the pangenome tree could basically distinguish three different species in the RSSC, just like the core-genome and whole-genome tree mentioned above. The pangenome phylogenetic analysis will facilitate the evolutionary analyses of species phylogeny reconstruction. These different methods could be useful in exploring the diversity of the RSSC in different ways.

### Distribution of VF Genes in Each Strain and the RSSC Pangenome

**Figure 4:**
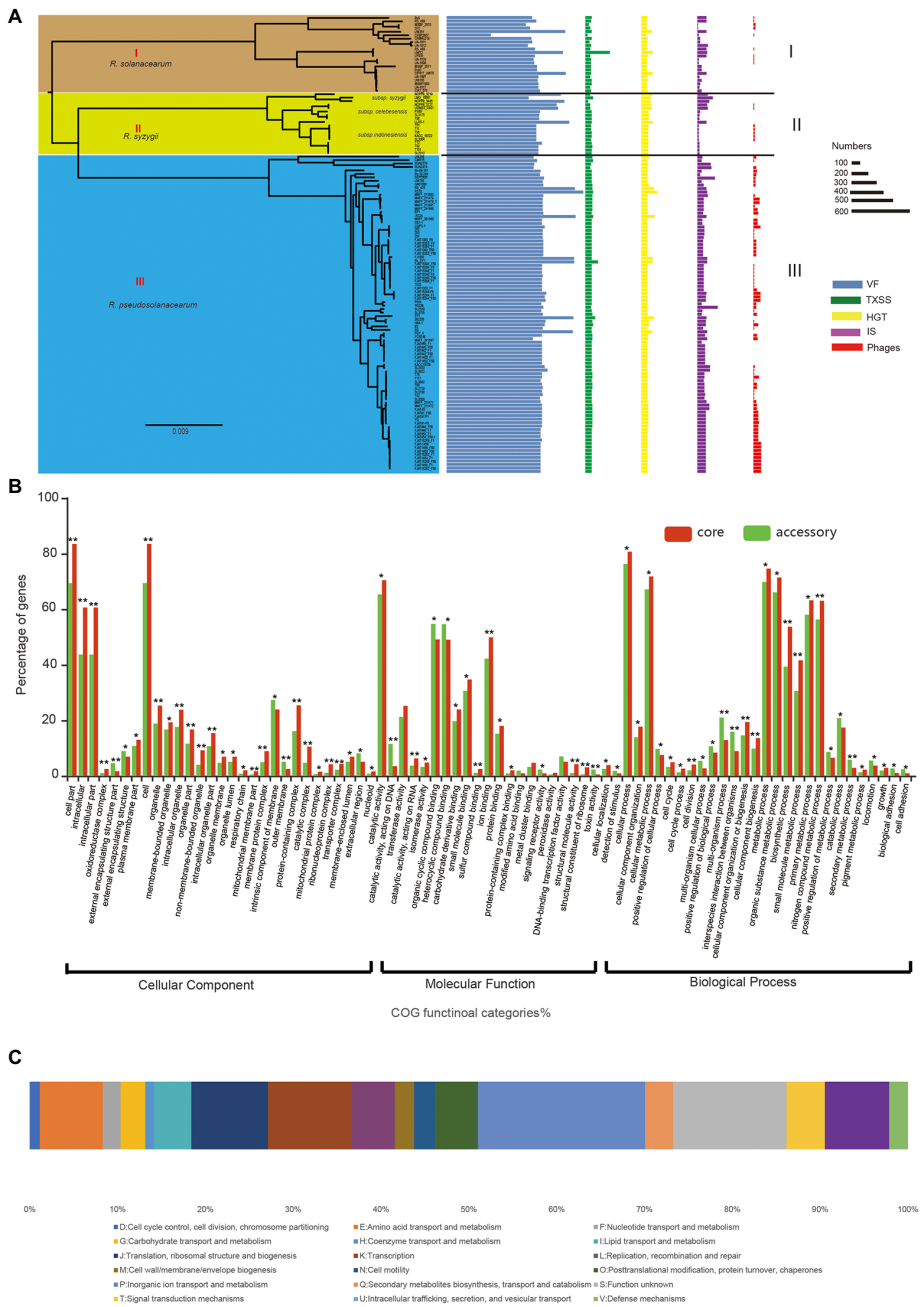
The pathogenicity and evolution of RSSC. **(A)** Distribution of virulence factor (VF), TXSS, horizontal gene transfer (HGT), insertion sequence (IS), and Phages in each strain of RSSC. The different groups were associated with that in the core genome tree (Figure 3A). **(B)** Distribution of GO functions in the core genome (red) and accessory genome (green). *t test *p* < 0.05; **t test *p* < 0.01. **(C)** Distribution of clusters of orthologous groups of proteins (COG) categories for HGT gene families.

Identification of virulent proteins can benefit the understanding of the mechanism of virulence and in drug design. The RSSC has a wide range of genetic variation, and its pangenome is relatively large, with more than 30,000 genes, but the core genome is very small, accounting for only 3.8% of the whole pangenome. From the perspectives of host range, pathogenicity, physiological characteristics, and geographical distribution, the RSSC is a highly diverse plant pathogen, and some strains have specificity to certain hosts (Genin and Denny, 2012; Ailloud et al., 2015; Guarischi-Sousa et al., 2016; Cho et al., 2019). Thus, the huge dispensable genomes of RSSC could be conducive to its adaptation to different environments or hosts. To get insight into the pathogenic potential of the RSSC, the current study explored the virulent genes in each strain as well as in the pangenome. A total of 62,144 VF genes were detected in 131 RSSC strains, with an average of 474 VF genes per strain (Figure 4A). Strain RS10 had the highest number of VFs at 792, while strain CFBP2957 had the lowest number of VFs at 257, and these VFs accounted for 15.1% and 7.6% of their total genes, respectively. Furthermore, we detected the distribution of VFs in the RSSC pangenome, and found 354, 1,949, and 596 VF genes in the core genome, accessory genome and unique genes, respectively. The core VFs were mainly associated with adherence, regulation, and iron uptake, and accounted for 50.0% (177 out of 354), which were the fundamental and important functions in the pathogenicity of *R. solanacearum*. For example, we have found 22 lateral/polar flagella- and Type IV pilus-related genes (such as *flgA*, *fliG*, *pliC*, *cheY*, and *scrC*), which played a great role in the flagellum-driven swimming motility and type IV pili-driven twitching motility and benefit the ecological fitness and virulence of *R. solanacearum* (Kang et al., 2002; Tans-Kersten et al., 2004; Genin and Denny, 2012). In addition, 37 core VF genes related to the synthesis of polysaccharides (including EPS, LPS, LOS, and CPS) were also detected. These genes could be involved in the process of immune evasion, since EPS is considered to be an abundant and indispensable virulence factor of *R. solanacearum* (Schell, 2000; Annett et al., 2011). Iron uptake systems can promote the pathogenesis of microorganisms and play an important role in iron competition at the microbiome level and plant protection (Gu et al., 2020; Klebba et al., 2021). In the core VFs of the RSSC, we detected 50 genes related to iron uptake. For example, ferric uptake regulator (*fur*) is a transcriptional regulator found in many Gram-positive and Gram-negative bacteria and is considered to control iron uptake and storage in response to iron availability, thus playing a key role in maintaining iron homeostasis (Andrews et al., 2003; Florent et al., 2016). Because the core VFs could cover nearly all strains and are the essential pathogenicity factors, they have become effective prevention targets for the RSSC with high diversity. The accessory VFs were mainly involved in the secretion system, adherence, iron uptake, and regulation, accounting for 66.6% (1,299 out of 1,949) of all accessory VFs. Among the secretion system genes, most (80.0%, 629 out of 786) belonged to T3SS, indicating their determinant role in the pathogenicity of the RSSC (Angot et al., 2006; Huilan et al., 2020). T3SS can inject effector proteins into the cytoplasm of host cells, causing disease in susceptible plants or hypersensitivity in resistant plants, and it also plays a biological role throughout the disease cycle (Jacobs et al., 2012; Hikichi et al., 2017). MprA/MprB is a two-component system of stress response, which is considered to directly regulate the expression of the sigma factors *sigB* and *sigE* in *Mycobacterium tuberculosis* (He et al., 2006), which may also be conducive to the stress adaptation of the RSSC. DevR is a response regulator of the two-component system DevR/DevS of *M. tuberculosis* and is considered to be essential to bacterial adaptation (Sharma et al., 2019; Kumari et al., 2020). In the accessory VFs, we found that four accessory families could be associated with DevR, which would be helpful to study the adaptation of *R. solanacearum* to different hosts and environments. The main VFs in the unique genes were secretion system, adherence, toxin, and immune evasion, accounting for 81.5% of all unique VFs. UW386, CMR15, Rs5, and RSCM all had more than 30 VFs, while a total of 70 strains did not have any unique VFs. AlgU is a stress-related sigma factor that actively regulates many factors involved in the production of alginate and has the function of inhibiting CRISPR-Cas immunity (Hay et al., 2014; Borges et al., 2020). AlgU has also been found to control the biosynthesis of exopolysaccharide and is a key determinant of the ability of *Pseudomonas fluorescens* to survive under dry conditions and high osmotic pressure (Schnider-Keel et al., 2001). Among the 131 strains in this study, AlgU was only found in YC40-M isolated from *Rhizoma kaempferiae*, which could be useful in the study of the host specificity of *R. pseudosolanacearum* in the next step (Prokchorchik et al., 2020). IBSBF1503 (*R. solanacearum*) was isolated from cucumber and belonged to phylotype II (Ailloud et al., 2015). It was not pathogenic to banana (NPB), but exhibited pathogenicity to cucurbits (Florent et al., 2016). Hemolysin secretion protein HlyB was the only unique VF detected in this strain, which functioned as an adenosine triphosphate (ATP)-binding cassette (ABC) transporter to enable the bacteria to secrete toxins at the expense of ATP hydrolysis (Gentschev et al., 2002; Zhou et al., 2018). In addition, 20 strains had single unique VFs, and 30 strains had fewer than 10 unique VFs, which may be related to their pathogenicity and host specificity. In addition, we statistically analyzed the distribution of VF in different groups of the RSSC, and found certain differences among the three species. The average number of VFs in *R. solanacearum*, *R. syzygii*, and *R. pseudosolanacearum* was 522, 566, and 567, respectively, with differences of approximately 9%. These differences in the VFs in three species might be the reason for the diverse pathogenicity of the RSSC. Therefore, the analysis of VF in each strain and pangenome can provide a new resource for the study of pathogenicity and host specificity of the RSSC (Supplementary Table S3).

### Evolution of Core and Accessory Genomes in RSSC

To identify the key functional gene changes in RSSC, 1,263 core gene families (single-copy gene families) and 2,748 accessory gene families, which covered more than 84 strains in this study, were selected for characterizing the evolutionary changes by measuring the ratio of non-synonymous to synonymous substitution rates (*dN/dS*). We found that the *dN/dS* for all the core gene families and 98.7% of accessory gene families (2,713) was less than 1, with an average of 0.09 and 0.18, respectively (Supplementary Table S4). Most core genomes (99.6%) and accessory genomes (91.3%) showed ratios less than 0.25, which strongly suggested purifying selection in *R. solanacearum*. For example, Dps (*dN/dS* = 0.03) is a non-specific DNA binding protein, which is very important for the stress survival of many bacteria (Smith, 2004). It was found that Dps also played an important role in oxidative stress tolerance, colonization and virulence of *R. solanacearum* (Colburn-Clifford et al., 2010). CheY (*dN/dS* = 0.13) is a diffusive cytoplasmic response regulator that interacts with flagellum movement to change its rotation direction and plays a key role in bacterial chemotaxis (Corral et al., 2020; Antani et al., 2021). In addition, the *dN/dS* for 36 genes were more than 1 in the accessory genome; thus, these genes were identified as positively selected. For example, GlnQ (*dN/dS* = 3.44) is a glutamine transporter and plays an important role in fibronectin adhesion and virulence of Group B *Streptococci* (Tamura et al., 2002). The protein is considered to be involved in a crucial step in glutamine metabolism, which is essential for the adaptability and virulence of various bacteria (Härtel et al., 2011). Also, we detected GO functions for core and select accessory gene families and found that both core and select accessory genes were enriched in cellular process, metabolic process, and catalytic activity (Figure 4B). The GO categories of oxidoreductase complex, organelle, transporter complex, and cell periphery showed obvious differences between core and accessory genes (value of *p* < 0.05). Meanwhile, genes involved in the intracellular, external encapsulating structure part, cell part, membrane part, and protein-containing complex exhibited significantly stronger evolution constraints than genes involved in other functions (value of *p* < 0.01), which demonstrated that core and accessory genes faced different selection pressures during the evolution of the RSSC.

### Macromolecular Secretion Systems Reflect the Pathogenic Potential of RSSC

Bacteria can secrete proteins into the extracellular environment or even inject them into adjacent cells with the help of a variety of secretion systems, which are essential for bacterial adaptation and pathogens (Maffei et al., 2017). To explore the macromolecular secretion systems in the RSSC, we have detected the distribution and organizations of all types of secretion systems using MacSyFinder software (Abby et al., 2015). Flagellum, T1SS, T2SS, T3SS, T5SS (including T5aSS, T5bSS, and T5cSS), T6SS, T4P, and the tight adherence (Tad) pilus were widespread in 131 strains, and pT4SSt and T4SSt were sporadically distributed in several strains (Supplementary Table S5). T1SS played an important role in the export of exogenous and toxic compounds, including antibiotics, VFs and adhesion molecules (Alav et al., 2021). The results showed that there were two T1SS gene clusters in 102 of 131 genomes and one T1SS gene cluster in 28 genomes, while no T1SS gene cluster was found in strain CFBP2957. Similarly, T2SS (130 of 131), T3SS (129 of 131), T4P (130 of 131), and Tad (128 of 131) were present in almost all the strains, suggesting that the existence of these secretion systems may represent the general characteristics of the RSSC and play a key role in their genomic plasticity and pathogenicity (Landry et al., 2020). For example, T4P is thought to be necessary for the natural transformation and virulence of *R. solanacearum* adhering to multiple surfaces (Kang et al., 2002). T4SS plays an important role in protein secretion and binding, and has the potential to be used as a tool for genetic modification (Low et al., 2014). Protein secretion-related T4SS has been found in 20 strains, mainly concentrated in *R. pseudosolanacearum* (80%, 16 out of 20), which could be related with their pathogenicity. For the different groups of the RSSC, there were also differences in quantity of genes related to these macromolecular secretion systems. We found that *R. solanacearum* had a minimum of 33 and a maximum of 360 with an average of 92, while *R. syzygii* had a minimum of 42 and a maximum of 109 with an average of 87, and *R. pseudosolanacearum* had a minimum of 74 and a maximum of 190 with an average of 100. In *R. solanacearum*, five strains (22.7%) did not have T6SS in their genomes, while almost all strains in the other two species were detected to have T6SS. In the RSSC, T6SS was considered to play an important role in the virulence of different plants such as tomato and eggplant. It was similar for T5cSS, which occurred in fewer strains of *R. solanacearum* (1/3 had no T5cSS), but was present in almost all strains of *R. syzygii* and *R. pseudosolanacearum*. The presence or absence of T5SS and T6SS might result in different pathogenic capacities of three RSSC species. Therefore, the distribution of macromolecular secretion system in the RSSC are highly diverse, and more research is needed to confirm their functions and potential role in pathogenicity.

### MGEs Mediated Genomic Plasticity of the RSSC

MGEs are highly diverse, and include phages, plasmids, GIs, and IS, which mediate the acquisition of DNA and facilitate the spread of gene modules of different evolution origins (Frost et al., 2005; Flores-Ríos et al., 2019). In this study, the distribution of prophages, GIs, and ISs had been detected in each genome of the RSSC (Supplementary Table S6). Prophages are lysogenic phages that integrate into the genomes of host bacterium and participate in replication. They can have a significant impact on bacterial genomes and phenotypes, leading to the emergence and diversification of strains that increase virulence or antibiotic resistance (Akhter et al., 2012). A total of 4,113 prophages were found in 131 genomes, with an average of 31.4. We found that the number of prophages in three groups differed. *R. pseudosolanacearum* had the largest number with an average of 44.87, and *R. solanacearum* and *R. syzygii* had an average of 6.72 and 9.83, respectively, suggesting high genomic plasticity of the RSSC. GIs are clusters of genes in the bacterial genome that appear to be acquired through HGT and may confer evolutionary advantages that allow for adaptation to new environments or new pathogenicity (Hacker and Kaper, 2000; Juhas et al., 2009). ISs were thought to be important in the organization and evolution of prokaryotic genomes (Siguier et al., 2014). In the RSSC, ISs were found to play a key role in virulence evolution and genomic plasticity (Gonçalves et al., 2020a). Here, we obtained a total of 13,425 ISs, with an average of 102. There was an obvious contrast between the different species of the RSSC, the average number was 75, 74, and 115 for *R. solanacearum*, *R. syzygii*, and *R. pseudosolanacearum*, respectively (Figure 4A). Since IS-mediated genome rearrangement and reduction can facilitate pathogen evolution by inserting and deleting DNA fragments, the different distribution of ISs could lead to the diversity evolution of these species. In this study, we also investigated the distribution of GIs in the pangenome level, which could reveal the HGTs and genome evolution among these RSSC strains. A total of 30,395 (92.2%) gene families were detected to have GIs in the pangenome, including 4,594 phages and 29 metal resistance proteins. In 109 strains, there were 15 different types of *Ralstonia* phages (e.g., phiRSA1, RSS20, and RSM3) and two types of *Burkholderia* phages (BcepMu and phiE255). These phages had a broad host range and could infect a variety of host strains, especially *Ralstonia* phage phiRSA1, which was considered to be highly pathogenic and could infect more *R. solanacearum* strains of different races or biovars (Yamada et al., 2007; Fujiwara et al., 2008). Additionally, phiRSA1 was the largest (3,547) phage in the pangenome of RSSC, accounting for 77.2% of these phages, and covered 77 strains, which occurred primarily in the *R. pseudosolanacearum* (81.8%, 63 out of 77 strains). Twenty-seven *Burkholderia* phages were detected in six different strains, indicating that there were potential HGT events from other *Burkholderiaceae* species to RSSC, which could benefit their environmental adaptation and pathogenicity. The metal resistance gene was responsive to the induction of heavy metals in bacteria (Mădălina et al., 2016), and 18 metal resistance proteins were found in 14 strains at last, which could be related to the different metal resistance of RSSC. For example, the chromium ion transporter (CHR) superfamily protein ChrA was found to be involved in chromate ion efflux from the cytoplasm, thus leading to chromate resistance (Pradhan et al., 2016), which was concentrated in the strains of *R. syzygii* in this study. Thus far, the metal resistance in the RSSC was limited, but further studies remained need to explore this in the future. The obvious differences in the distribution of MGEs among the three species might be associated with their different pathogenicity and potential host specificity, which required further research for these *Ralstonia* species in the future.

### Detection and Functional Landscape of HGT Candidates

HGT refers to the process of transferring genetic information in organisms by introducing new genes into an existing genomes, helping recipient organisms bypass point mutations and recombination to create new genes, so as to accelerate genome innovation and evolution (Soucy et al., 2015; Husnik and Mccutcheon, 2018). In this study, a total of 13,363 genes were detected in 131 strains *via* HGT, with an average of 102 HGT genes in each genome (Figure 4A; Supplementary Table S6). For the different species of the RSSC, the average number of HGTs in *R. solanacearum*, *R. syzygii*, and *R. pseudosolanacearum* was 96, 118, and 100, respectively. RS10 had 237 HGT genes, which was the largest number among these strains. Besides that, there were 19 strains contained more than 110 HGT genes. T98 and SL3175 were located in group I (*R. solanacearum*) of the core-genome tree and cluster b of the pangenome tree, with the same number of dispensable genes (76.4%), genome size, and VFs. A 60-kb-long Integrative and Conjugative Element (ICE) was also found in their chromosomes, which may prove that they have a highly similar evolution distances (Gonçalves et al., 2020b). UW386, 362,200, and GMI1000 were distributed in group III (*R. pseudosolanacearum*), but showed significant differences in both of the core-genome and whole-genome tree. The phylogenetic diversity of these five strains suggested that HGT may play an important role in the evolution and genomic diverse of RSSC. Novel traits that evolve through HGT can lead to the exploitation of new niches, prompting an adaptive radiation to exploit new resources without competition (Soucy et al., 2015). We found that HGT genes of RSSC were mainly from *Burkholderiales* (81.2%), *Oxalobacteraceae* (8.6%), and *Alcaligenaceae* (3.2%). HGT genes that directly affect the competition and adaptation of similar species are considered to have the highest transfer efficiency among bacterial strains (Yerrapragada et al., 2009). In addition to 1,577 HGT genes with poor characteristics (function unknown or no homologs identified), we have found 8,139 HGT gene families (72.1% of all HGTs) in the accessory genome, which were enriched in “P: Inorganic ion transport and metabolism,” “K: Transcription” and “E: Amino acid transport and metabolism” (Figure 4C). It is thought that HGT is common in closely related species with highly similar genomic characteristics, especially for accessory genes that can be obtained through HGT among bacterial species (Golicz et al., 2020). Only few HGT genes were assigned to the unique genes (approximately 0.69%, 78 out of 11,289), which were mainly involved in the functions of “U: Intracellular trafficking, secretion, and vesicular transport,” “L: Replication, recombination and repair,” and “E: Amino acid transport and metabolism.” We found that HGT also contributed to the core genomes of the RSSC. A total of 3,072 core gene families had potential HGT events during the evolution of RSSC, which were enriched in “P: Inorganic ion transport and metabolism,” “U: Intracellular trafficking, secretion, and vesicular transport” and “J: Translation, ribosomal structure and biogenesis.” Evidence from phylogenetic comparisons in other studies suggests that horizontal genetic exchange between RSSC plays an important role in their evolution (Guidot et al., 2009; Coupat-Goutaland et al., 2010). These genes benefit the energetic balance through inorganic ion transport or improve the defense capability of RSSC under adverse circumstances.

## Conclusion

*R. solanacearum* is one of the most devastating plant pathogens worldwide. It encompasses a variety of ecotypes and species, which brings great difficulties to its accurate taxonomy, and many strains are still misclassified in NCBI. Meanwhile, due to the high complexity and diversity of the RSSC, exploring its pathogenicity and host specificity remains a great challenge. This study evaluated the genetic diversity and evolutionary dynamics of the RSSC based on pangenome analyses to comprehensively understand the highly diverse phylogeny and pathogenicity of RSSC and facilitate exploring the differences among the three distinct species. Although the new nomenclatures of RSSC have been developed for several years, RSSC still has many incorrect classifications and is often referred to as *R. solanacearum*, and many strains are difficult to be identified by their correct species name. Based on comparative genomic analysis of 131 complete genomes, the RSSC exhibited an open pangenome with 30,763 gene families, which indicated extensive genetic diversity and increased the knowledge of its gene repertoire, to be used with newly sequenced strains in the future. It was considered that the size of core and accessory genomes was associated with the lifestyle (Brockhurst et al., 2019). The large dispensable genome (96.3%) was involved in the functions of replication, recombination and repair, which could benefit RSSC in surviving in different environments or hosts. The phylogenetic relationships of the RSSC were investigated at the levels of the core genome, pangenome and whole genome. We found that the three trees were divergent, which showed the evolutionary dynamics among these strains and indicated that the diverse and large proportion of accessory genome and unique genes could affect the evolution of RSSC. The ANI values of 131 strains were significantly different, which could be divided into three groups, indicating that there were great differences among the three different species at the genomic level. The results were consistent with the new nomenclatures of RSSC in previous studies (Remenant et al., 2010; Safni et al., 2014; Prior et al., 2016), and helped many strains (78.6%, 103 out of 131 strains) to get the correct taxonomy. Furthermore, we found that the core-genome phylogenetic analysis could distinguish three species clearly, and was consistent with the whole-genome analysis, thus, could benefit the classification of the RSSC. To accurately classify three distinct species of RSSC, especially the subspecies of *R. syzygii*, we also downloaded eight genomes of *R. syzygii* and *R. pseudosolanacearum*. In this study, we found 12 strains (labeled as *R. solanacearum* in NCBI) had a closer evolutionary relationship with *R. syzygii* in Group II of both core-genome and whole-genome trees. According to the known subspecies taxonomy, we have attempted to identify the subspecies names of several *R. syzygii* strains. More research and experiments are still needed to verify the classifications of these subspecies in the future. Different selective pressures act on diverse functions in the RSSC, with the core genes and most accessory genes experiencing purifying selection. Our study provides evidence from phylogenetic comparisons and HGT detection that horizontal genetic exchange mainly from *Burkholderiales* could play a significant role in the evolution and that HGT is a driver that shapes the genetic diversity of the RSSC genomes. In addition, the variety distribution of VFs, HGTs and MGEs among three species indicated the high genomic plastic and pathogenicity. Further functional analysis of HGTs, macromolecular secretion systems and MGEs has provided rich sources for the study of the diverse pathogenicity and host specificity of RSSC in the future.

## Data Availability Statement

The original contributions presented in the study are included in the article/Supplementary Material, and further inquiries can be directed to the corresponding authors.

## Author Contributions

AY, MR, and RG conceived the project. RG, LC, CC, ZL, XW, ZX, ZH, and QF performed the sequences and other data analysis. RG, DL, CL, ZC, ZZ, and CJ conducted the bioinformatics analyses. RG, MR, and AY prepared the manuscript. All authors contributed to the article and approved the submitted version.

## Funding

This work was supported by Tobacco Genome Project of State Tobacco Monopoly Administration [110201901015 (JY-02)] and Agricultural Science and Technology Innovation Program (ASTIP–TRIC01).

## Conflict of Interest

CC is employed by Shandong Rizhao Tobacco Company Ltd.

The remaining authors declare that the research was conducted in the absence of any commercial or financial relationships that could be construed as a potential conflict of interest.

## Publisher’s Note

All claims expressed in this article are solely those of the authors and do not necessarily represent those of their affiliated organizations, or those of the publisher, the editors and the reviewers. Any product that may be evaluated in this article, or claim that may be made by its manufacturer, is not guaranteed or endorsed by the publisher.

## Footnotes

^1^ https://github.com/Jtrachsel/gifrop

## Supplementary Material

Supplementary Figure S1 The different sizes of the pangenome of RSSC and sub-pangenomes of three species. **(A)** The composition of core, accessory and unique genes in the pangenome based on 131 strains of RSSC. **(B)** Size of sub-pangenome based on 22 strains of *R. solanacearum* (Group I). **(C)** Size of sub-pangenome based on 18 strains of *R. syzygii* (Group II), which had the largest core genomes than other two groups. **(D)** Size of sub-pangenome based on 91 strains for *R. pseudosolanacearum* (Group III), with the largest unique genes than groups I and II.

Supplementary Figure S2 Phylogenetic tree based on the pangenomes of RSSC. Pangenome tree could be divided into four groups (red letters A–D), the gene abundance of core, accessory and unique with red, green and purple bar on the top. The right rectangles with different colors were associated with the groups in the whole and core genome tree in Figure 3.

Supplementary Table S1 Genome statistics of the sequenced RSSC strains in this study.

Supplementary Table S2 Distributions of the core, accessory, and unique genes in RSSC pangenome and sub-pangenome, with the group numbers in the core-genome, whole genome and pangenome trees.

Supplementary Table S3 Summary of VFs in RSSC pangenome.

Supplementary Table S4 The ratio of non-synonymous to synonymous substitution rates (*dN*/*dS*) in the core and selected accessory genes of RSSC.

Supplementary Table S5 Distributions of TXSS gene clusters in each strain of RSSC.

Supplementary Table S6 Summary of HGT, IS, Phages, and GIs in each strain of RSSC.
