# Supplementary figures and images for: Comprehensive Analysis Reveals the Genetic and Pathogenic Diversity of Ralstonia solanacearum Species Complex and Benefits Its Taxonomic Classification
Source: Front Microbiol. 2022 May 6;13:854792. doi: 10.3389/fmicb.2022.854792 (PMC9121018; doi:10.3389/fmicb.2022.854792)

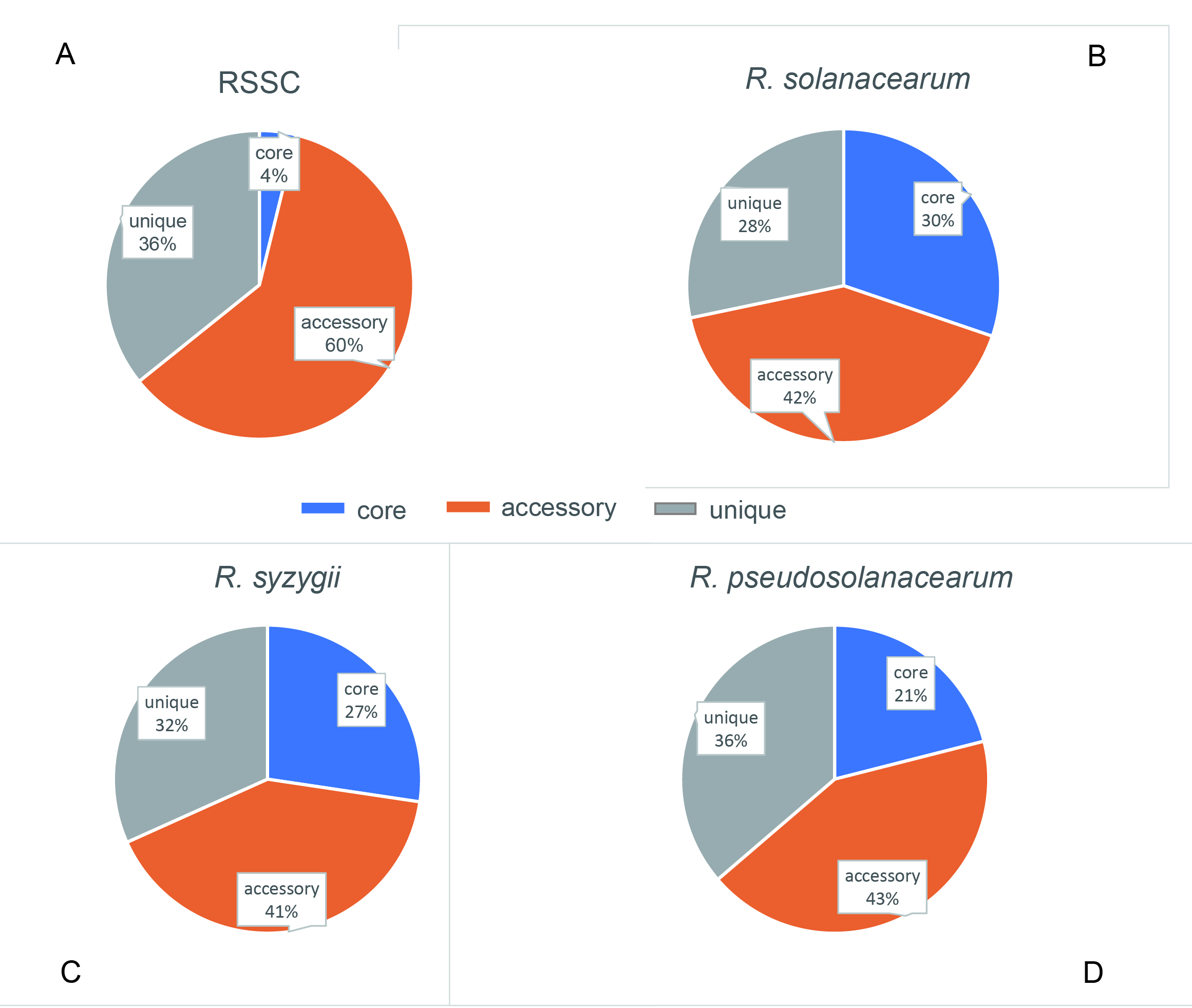

Supplement: Supplementary Figure S1 — The different sizes of the pangenome of RSSC and sub-pangenomes of three species. (A) The composition of core, accessory and unique genes in the pangenome based on 131 strains of RSSC. (B) Size of sub-pangenome based on 22 strains of R. solanacearum (Group I). (C) Size of sub-pangenome based on 18 strains of R. syzygii (Group II), which had the largest core genomes than other two groups. (D) Size of sub-pangenome based on 91 strains for R. pseudosolanacearum (Group III), with the largest unique genes than groups I and II. [file Image_1.JPEG]

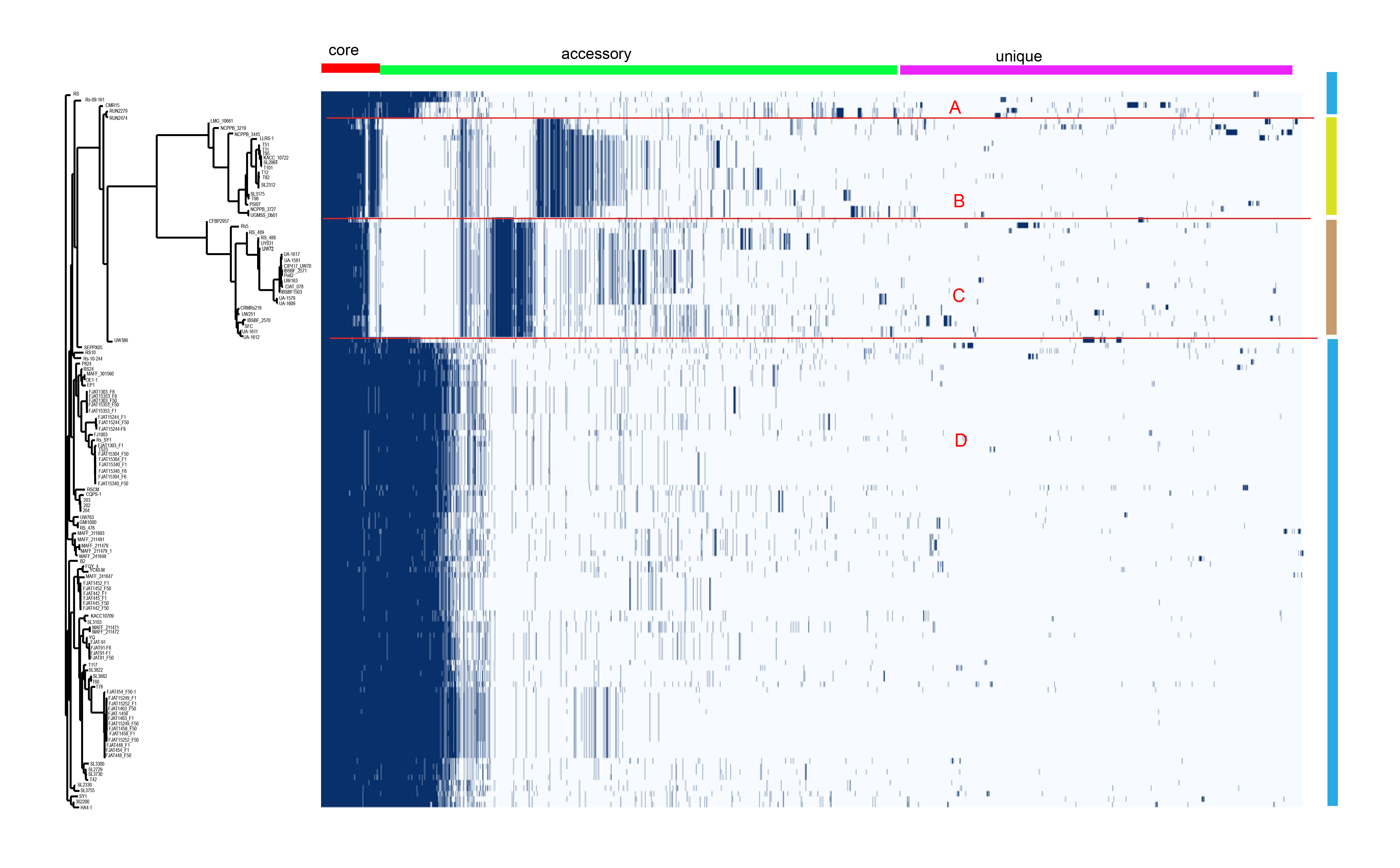

Supplement: Supplementary Figure S2 — Phylogenetic tree based on the pangenomes of RSSC. Pangenome tree could be divided into four groups (red letters A–D), the gene abundance of core, accessory and unique with red, green and purple bar on the top. The right rectangles with different colors were associated with the groups in the whole and core genome tree in Figure 3. [file Image_2.JPEG]
